# The mediating role of mindfulness and psychological resilience in the relationship between internalized stigma and caregiver burden among parents of children with Autism Spectrum Disorder

**DOI:** 10.3389/fpsyg.2026.1751960

**Published:** 2026-02-17

**Authors:** Ayfer Öztürk

**Affiliations:** Department of Psychiatric Nursing, Faculty of Health Sciences, Bartin University, Bartin, Türkiye

**Keywords:** Autism Spectrum Disorder (ASD), caregiver burden, mindfulness, parents, resilience, stigma

## Abstract

**Background:**

Stigma surrounding Autism Spectrum Disorder (ASD) is prevalent, but limited studies have examined its negative psychological effects on parents of children with ASD and the potential protective factors in this family context. Moreover, no research has examined the mediating role of factors such as psychological resilience and mindfulness, that we think may consider potential buffers in the relationship between internalized stigma and caregiver burden which one of its negative consequences for these parents. The aim of this study is to investigate the mediating role of psychological resilience and mindfulness in the relationship between perceived caregiver burden and internalized stigma in parents of children with ASD and to provide a theoretical basis for studies aimed at reducing internalized stigma and caregiver burden in these parents.

**Methods:**

The study was conducted between March 2023 and January 2025 with a sample of 138 parents of children diagnosed with ASD receiving services from special education and rehabilitation centers. Data analysis included descriptive statistics, Pearson correlation, multiple linear regression, and Structural Equation Modeling (SEM) to assess mediation effects. For normally distributed data, Independent Samples *t*-test and One-Way ANOVA were applied to compare group differences.

**Results:**

The study found that parents had average total scores of 14.39 for resilience, 65.97 for internalized stigma, 55.59 for caregiver burden, and 52.93 for mindfulness. Caregiver burden was significantly higher among parents aged 20–25, those with lower income, and female parents. Additionally, having another child with a disability was associated with higher internalized stigma and lower resilience. Internalized stigma was significantly correlated with resilience, caregiver burden, and mindfulness (*p* < 0.001 for all). Psychological resilience (*β* = 0.174) and mindfulness (*β* = 0.254) were found to have significant indirect effects and partially mediated the relationship between internalized stigma and caregiver burden.

**Conclusion:**

These research findings indicate mindfulness and psychological flexibility are factors contributing to resilience against caregiver burden. Future interventions to reduce burden in caregivers of children with ASD might be improved by the inclusion of resilience and mindfulness training programs.

## Highlights

- Internalized stigma adversely affects parents of children with Autism Spectrum Disorder.
- One of the negative effects of stigma on parents of children with ASD is that it increases their perception of caregiver burden.
- Stigma surrounding ASD is common; however, there are limited studies on the caregiver burden perceived by parents of children with ASD and the potential protective factors in this family context.
- Resilience and mindfulness are generally regarded as important compensatory factors that buffer individual psychological responses to challenging conditions, facilitate coping with such challenges, and serve as moderators in the relationship between stigma and its psychological consequences.
- No research has been identified that specifically explores the connection between internalized stigma and adverse outcomes, such as heightened caregiver burden, among parents of children with ASD, nor examines the potential buffering effects of mindfulness and psychological resilience on these associations.
- Psychological resilience and mindfulness moderate the impact of internalized stigma perceived by parents of children with ASD on their perception of caregiver burden.
- It is important to help cultivate awareness among parents of children with ASD and enhance their psychological resilience.

## 1 Introduction

Autism Spectrum Disorder (ASD) is a neurodevelopmental disorder characterized by deficits in social reciprocity, delays in communication skills, and the presence of repetitive behaviors that may violate social norms. It is more prevalent in males, with a ratio of approximately 4:1. In addition, children with ASD often face stigma due to stereotypes, misconceptions, and discrimination from mainstream society (Liao et al., 2019). Despite recent studies indicating that individuals with autism often mask their differences to integrate into society and maintain interpersonal relationships, stigma surrounding autism persists (Botha et al., 2022; Perry et al., 2022; Ma et al., 2023). Stigma not only impacts children with ASD, but also extends to individuals who are close to them, particularly their parents. Parents of children with autism experience sequential process of stigmatization which begins with negative stereotyping, loss of societal status, separation and isolation and culminates into discrimination (Ma et al., 2023; Patra and Kumar, 2019; Salleh et al., 2020; Öz et al., 2020; Oduyemi et al., 2021). A previous study evaluating stigmatization in families with children with ASD found that society perceived ASD-specific behavioral patterns as “weird,” leading to hurtful words and looks directed at both individuals with ASD and their families. This, in turn, restricted their social lives. Families also reported that, to avoid societal judgment, they preferred to interact only with close relatives and other families with disabled children, whom they believed would understand them and refrain from judgment (Karabekiroğlu et al., 2009). People who do not understand children’s behaviors with ASD probably would not associate them with mental conditions but question their caregivers instead. This may put extra pressure on caregivers and, thus, lead to autism-related stigmatization (Ma et al., 2023). In view of the prevalent and stigma against their children and themselves, parents of children with ASD may perceive their families as inferior to others and have self-stigmatizing thoughts about themselves (Chan and Lam, 2018).

The personal suffering experienced by a caregiver as a result of a family member’s illness is referred to as “burden.” In the literature, a distinction is commonly made between objective burden and subjective burden. Objective burden pertains to practical difficulties, such as disrupted family relationships, limitations in social, leisure, and work activities, and financial struggles. Subjective burden, on the other hand, relates to the caregiver’s psychological responses, including loss of hope, shattered dreams and expectations, depression, anxiety, and feelings of embarrassment in social situations (Picardi et al., 2018). Since most individuals with autism spectrum disorders continue to live in family homes into adulthood, the responsibility for assisting with activities of daily living often falls on parental caregivers. Providing continuous support to children with ASD who struggle with independently performing daily activities may increase the caregiver burden experienced by their aging parents (Marsack-Topolewski et al., 2021). Being a parent of a child with a neurodevelopmental disorder such as autism spectrum disorder requires making many correct moves from the moment of birth. Responsibilities such as ensuring the child’s personal care, meeting their special needs, assisting them in accomplishing various tasks, ensuring that their school life is not disrupted, and developing their social life skills fall on the caregiver. In fact, while trying to fulfill these responsibilities, parents may neglect their own lives and health. Therefore, parents, who are mostly in the caregiver position, may perceive this upbringing process as a heavy burden and, when they feel unable to continue supporting their child, become vulnerable to depression and anxiety disorders (Ceco and Çakın Memik, 2017). Moreover, the internalized stigma perceptions of parents of children with ASD can increase the caregiver burden they experience. While numerous studies focus on stigma experienced by parents of children with ASD, fewer studies have explored the internalized stigma perceptions and their relationship with caregivers’ burden (Gabra et al., 2021; Gümüş and Kaçan, 2024; Efe et al., 2024).

The concept of mindfulness has gained significant prominence in contemporary psychological discourse. One of the primary reasons for this is the growing recognition that conscious awareness plays a crucial role in addressing psychological challenges. Mindfulness emphasizes the importance of acknowledging and regulating one’s emotional experiences, rather than suppressing or attempting to control them. Emotion regulation, in conjunction with mindfulness, serves to mitigate emotional and physiological responses, thereby fostering healing and emotional well-being. Mindfulness may enable individuals to approach negative life experiences with open and accepting attitudes (Kabat-Zinn, 1994, 2003). Given the deleterious effects of internalized stigma on self-perception emotional well-being, and perceived caregiver burden it is crucial to identify ways to help parents of children with ASD build resistance and resilience against stigma and protect themselves from the adverse psychological outcomes. According to Chan’s et al. (2018) theoretical model of stigma resistance, one potential way to protect stigmatized individuals from stigma-related harm is to cultivate mindfulness. Mindfulness has been linked to fewer emotional symptoms and mental health issues, according to earlier cross-sectional correlational research (Brown-Iannuzzi et al., 2014; Wang et al., 2024). Further evidence from intervention research has shown that mindfulness improves coping strategies and stigma resistance across various stigmatized populations (Tang et al., 2021). It is unclear, nevertheless, exactly how mindfulness impacts parental internalized stigma in children with autism (Wang et al., 2024). To date, there has been a limited number of studies investigating mindfulness as a protective factor against stigma in parents of children with ASD. The existing findings suggest that mindfulness may help parents alleviate negative thoughts and emotions associated with their stigmatized status (Chan et al., 2018; Wang et al., 2024; Yip and Chan, 2022).

Resilience is a must for parents of children with ASD. Psychological resilience is an internal factor that can improve the mechanism of coping with difficulties. Many parents of children with ASD have to deal with constant trials and associated difficulties and are therefore usually resilient. Resilience helps maintain personal wellbeing and function (Salleh et al., 2024). Nevertheless, to date, only a limited number of studies have examined the relationship between caregiver burden, stigma, and psychological resilience among parents of children with ASD (Salleh et al., 2022, 2024). In a study conducted by Salleh et al. (2022), low caregiver burden was reported to be associated with high resilience. In another study conducted by Salleh et al. (2024), the potential protective effect of psychological resilience against stigma among parents of children with ASD was reported. The relationship between psychological resilience, mindfulness, and caregiver burden has been examined in various sample groups, particularly among caregivers of individuals with chronic illnesses. Findings from these studies have reported that mindfulness and psychological resilience are contributing factors to resilience against caregiver burden (Klietz et al., 2020; Tan et al., 2023; Erdoğan Yüce et al., 2024; Saragih et al., 2024). However, no research has been identified that specifically explores the connection between internalized stigma and adverse outcomes, such as heightened caregiver burden, among parents of children with ASD, nor examines the potential buffering effects of mindfulness and psychological resilience on these associations.

To summarize, although stigma attached to ASD is prevalent, few studies have examined its impact on caregiver burden among parents of children with ASD, as well as the potential protective factors within this family context. Moreover, no research has specifically investigated the potential effect of internalized stigma on caregiver burden, or whether mindfulness and psychological resilience mediate the relationship between stigma and caregiver burden in this population. A limited number of previous studies have reported a positive association between stigma and caregiver burden among parents of children with ASD. Additionally, although not extensively studied, some evidence suggests that mindfulness may contribute to the development of resistance against stigma among these parents. While this has not been studied specifically in parents of children with ASD, previous research in different sample groups has shown that mindfulness and psychological resilience are contributing factors to resilience against caregiver burden.

Based on these findings, the present study aims to investigate the mediating role of two factors psychological resilience and mindfulness which are assumed to act as buffers in the relationship between internalized stigma and caregiver burden in parents of children with ASD. This study also seeks to provide a theoretical foundation for future interventions aimed at reducing internalized stigma and caregiver burden in this population.

**Figure 1:**
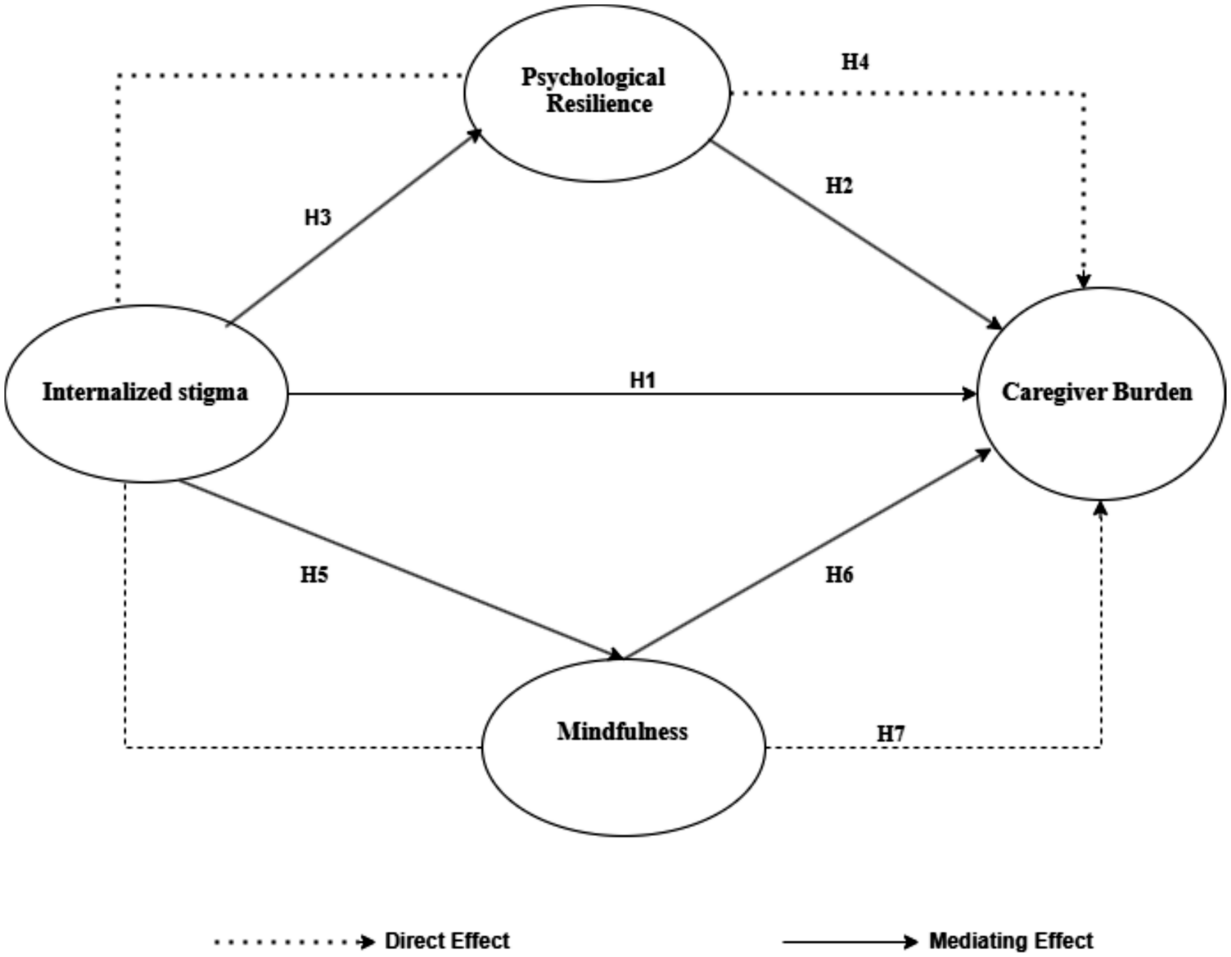
Hypothesized model.

Based on previous studies and the conceptual model outlined, the research model (Figure 1) was created and hypotheses were determined, with the idea that psychological resilience and mindfulness could act as a mediating variable in the relationship between internalized stigma and caregiver burden among parents of children with ASD.

### 1.1 Major hypotheses

H_1_: Internalized stigma positively and significantly predicts caregiver burden.

H_2_: Psychological resilience negatively and significantly predicts caregiver burden.

H_3_: Internalized stigma negatively and significantly predicts psychological resilience.

H_4_: Psychological resilience has a mediating role in the relationship between internalized stigma and caregiver burden.

H_5_: Internalized stigma negatively and significantly predicts mindfulness.

H_6_: Mindfulness negatively and significantly predicts caregiver burden.

H_7_: Mindfulness has a mediating role in the relationship between internalized stigma and caregiver burden.

## 2 Methods

### 2.1 Research design

A cross-sectional study design was employed in this investigation. The methodology aligns with the guidelines set forth in the Strengthening the Reporting of Observational Studies in Epidemiology (STROBE) checklist, ensuring rigorous and transparent reporting.

### 2.2 Participants and setting

This is a descriptive correlational study, using convenience sampling. The participants voluntarily agreed to participate in the study. The study was conducted between March 10, 2023, and January 10, 2025, in five Special Education and Rehabilitation Centers located in the central district of a province in Turkey. A total of 138 parents of children diagnosed with ASD who were receiving services from these centers participated in the study.

In Türkiye, children with autism spectrum disorder may receive educational support through different pathways, including mainstream schools with inclusive education support and Special Education and Rehabilitation Centers. The latter provide structured, individualized educational and therapeutic services and are commonly attended by children with more pronounced developmental needs. In the present study, participants were recruited exclusively from Special Education and Rehabilitation Centers, which should be considered when interpreting the findings. The severity of autism spectrum disorder and the presence of comorbid conditions in the children were not formally assessed in this study.

Data were collected using face-to-face interviews. The interviews were conducted in the waiting rooms of the institutions while parents were bringing their children for education. Each interview lasted approximately 15–20 min. The inclusion criteria for participation in the study were being a parent of a child diagnosed with ASD, being literate, and volunteering to participate in the research. Based on the study of Wolf et al. (2013), to determine the sample size in structural equation modeling, he introduced a ratio of 5–10 samples per variable (number of questions). According to the number of 29 questions, a sample size between 145 and 290 seems desirable. Since the study group included only parents of children diagnosed with autism and the research was conducted in five special education centers located in a single provincial city center, the data collection period was extended to reach a sufficient sample size. A total of 147 parents were initially asked to complete the questionnaire forms; however, this study excluded participants who did not respond to one or more questions, and the final analysis was conducted with 138 participants. Additionally, the sample size required for this study was determined using the G*Power 3.1.9 program. The study aimed to examine the relationships between internalized stigma, psychological resilience, mindfulness, and caregiver burden, as well as to test the mediating effects among these variables. In this context, multiple regression analysis was selected as the appropriate statistical method.

The following parameters were used for the power analysis: effect size = 0.2 (a small to medium effect according to Cohen’s guidelines), alpha level = 0.05 (indicating a 5% risk of Type I error), power (1−*β*) = 0.80 (indicating an 80% probability of correctly rejecting the null hypothesis), number of tested predictors = 2, and total number of predictors = 4. Based on these parameters, the required minimum sample size was calculated as 133. Therefore, the final sample size of 138 participants was considered sufficient for the analyses conducted in this study (Kang, 2021)^.^

### 2.3 Instruments

The data were collected by using the Participant Information Form, Parents’ Internalized Stigma of Mental Illness Scale, Zarit Caregiver Burden Scale, Brief Resilience Scale and Mindful Attention Awareness Scale.

#### 2.3.1 Participant information form

This questionnaire included a total of nine items aimed at collecting information from parents on variables that may be related to internalized stigma and caregiver burden. These items covered age, gender, income level, employment status, number of children, whether they have another child with a disability, the number of hours spent with their child per day, the gender of the child with ASD, and the duration (in months) of receiving services from the special education center.

#### 2.3.2 Parents’ Internalized Stigma of Mental Illness Scale (PISMIS)

Developed by Dikeç et al. (2020), the Parents’ Internalized stigmatization in Psychological Illness Scale was used in the study. The scale consisted of 29 items in a 4-point Likert design, based on self-reporting. The scale has the following 5 subscales; “alienation (Items: 1, 5, 8, 16, 17, 21),” “approval of stereotypes (Items: 26, 10, 18, 19, 23, 29),” “perceived discrimination (Items: 3, 15, 22, 25, 28),” “social withdrawal (Items: 4, 9, 11, 12, 13, 20),” “resistance to stigmatization (Items: 7, 14, 24, 26, 27).” The items on the scale are answered as “I strongly disagree” (1 point), “I disagree” (2 points), “I agree” (3 points), and “I strongly agree” (4 points). The items of the resistance to stigmatization subscale (7, 14, 24–26) are reversely coded. The total ISMI score obtained by adding the five subscales ranges from 29 to 116 and there is no cut-off score for the scale. Higher scores indicate that the individual’s internalized stigmatization is more severe in the negative direction. In this study, the total Cronbach Alpha value of the scale was calculated as 0.957.

#### 2.3.3 Zarit Caregiver Burden Scale (ZCBS)

The Caregiver’s Burden Scale is a data collection tool that was developed by Zarit et al. (1980) to evaluate the difficulties faced by caregivers and to uncover this situation. The adaptation studies of the scale into Turkish were conducted by İnci (2006). The scale has a Likert-type rating that ranged between 1 and 5 as “never,” “rarely,” “sometimes,” “often” and “almost always.” The scale, which can be completed either by the caregivers themselves or administered by researchers, consists of 19 items that assess the impact of caregiving on an individual’s life. There are no reverse-scored items on the scale. The items primarily focus on social and emotional aspects, and a higher total score indicates a greater level of distress experienced by the caregiver (Özlü et al., 2009). The version of the scale adapted to Turkish consisted of a single dimension. The internal consistency coefficient of the scale was found to be 0.95. In the present study, the total Cronbach’s Alpha value was calculated as 0.94.

#### 2.3.4 Brief Resilience Scale (BRS)

The scale was developed by Smith et al. (2008) and adapted into Turkish by Doğan (2015). The short form of this five-point Likert-type scale includes six items, ranging from “Not at all true” (1) to “Completely true” (5). Items 2, 4, and 6 are reverse-scored. A higher total score on the scale indicates a higher level of psychological resilience. The reliability coefficient of the original Turkish version was reported as 0.83. In the present study, after removing the first item due to low reliability, the Cronbach’s Alpha coefficient was calculated as 0.76.

#### 2.3.5 Mindful Attention Awareness Scale (MAAS)

The Scale was developed by Brown and Ryan (2003) and adapted into Turkish by Özyeşil et al. (2011). The scale consists of 15 items rated on a 6-point Likert scale and is designed to assess individuals’ tendencies to be aware of and attentive to present-moment experiences in daily life. Items are rated on a continuum from “almost always” to “almost never.” The scale has a unidimensional structure and does not contain a cut-off score. Higher scores reflect greater levels of mindfulness. In the Turkish adaptation study, the Cronbach’s Alpha coefficient was found to be 0.82. In the current study, the Cronbach’s Alpha coefficient was determined to be 0.93.

### 2.4 Statistical analysis

The data obtained in the study were analyzed using SPSS (Statistical Package for the Social Sciences) version 22.0 and AMOS Graphics software. Prior to the main analyses, a missing data analysis was conducted, and participants who had incomplete responses on any of the scales were excluded from the study. To evaluate the reliability of the measurement tools, Cronbach’s Alpha coefficients were calculated, and the results showed that the internal consistency of all scales was at an acceptable level. Descriptive statistics were used to analyze participants’ demographic characteristics (such as age, gender, income level, number of children, and employment status) as well as study-related variables (including the gender of the child with autism, the duration of receiving services from the special education center, whether another child had a disability, and the number of hours per day the parent spent with their child). To assess the normality of the data distribution, skewness and kurtosis values were examined. In this study, skewness values ranged between −0.027 and 0.274, and kurtosis values ranged between −0.674 and −1.390. According to Tabachnick et al. (2013), skewness and kurtosis values within the ±3 range indicate an acceptable level of normal distribution. Therefore, parametric tests were applied. An Independent Samples *t*-test was used to analyze differences between two groups, and One-Way ANOVA was used for comparisons among three or more groups. Before testing the structural model, Pearson correlation analysis was conducted to determine the relationships between the study variables. To test the proposed research model, Structural Equation Modeling (SEM) was performed using the maximum likelihood estimation method in AMOS 24.0. The mediation model was evaluated based on the criteria proposed by Baron and Kenny (1986), which involve the following steps: (1) assessing the effect of the independent variable on the dependent variable, (2) assessing the effect of the independent variable on the mediator variable, and (3) assessing the effect of the mediator variable on the dependent variable. Finally, the complete model was tested, and the mediating role of the variables was analyzed within the overall structure.

To assess potential common method variance, Harman’s single-factor test was conducted using exploratory factor analysis. The results showed that the first factor accounted for less than 50% of the total variance, indicating that common method variance was not a significant concern in this study. These findings suggest that the observed relationships among the study variables are not solely attributable to the measurement method.

## 3 Results

### 3.1 Participant characteristics

The study sample included 138 participants, of whom 109 (79.0%) were female and 29 (21.0%) were male. In terms of age distribution, 38 participants (25.5%) were aged 41 and above, 34 (24.6%) were between 36 and 40 years old, 33 (23.9%) were between 31 and 35 years old, 20 (14.5%) were between 26 and 30 years old, and 13 (9.4%) were between 20 and 25 years old. Most participants (*n* = 96, 69.6%) reported that they were not employed, while 42 (30.4%) indicated that they were employed. Nearly half of the participants (47.8%) had two children, and the majority (63.0%) reported a moderate income level. Among those with more than one child, 85 participants (73.27%) reported that none of their other children had a disability, whereas 31 (26.57%) indicated that they did. The majority of children diagnosed with ASD (65.2%) were male. Additionally, 84 participants (60.9%) reported spending six or more hours per day with their child (see Table 1). The average duration of receiving services from a special education center was 50.78 months, ranging from 1 to 228 months. No statistically significant association was found between the scale scores and the duration of special education service received (*p* > 0.05), (Table 1).

**Table 1:** Participant characteristics.

Variable	Category	*n*	%
Age	20–25	13	9.4
	26–30	20	14.5
	31–35	33	23.9
	36–40	34	24.6
	41 and above	38	27.5
Gender	Male	29	21.0
	Female	109	79.0
Employment	Yes	42	30.4
	No	96	69.6
Perceived income level	Low	39	28.3
	Medium	87	63.0
	High	12	8.7
Number of children	One	22	15.9
	Two	66	47.8
	Three	43	31.2
	Four or more	7	5.1
Disability in other children	Yes	31	26.57
	No	85	73.27
Child’s gender	Female	48	34.8
	Male	90	65.2
Time spent with child daily	1–2 h	5	3.6
	2–3 h	12	8.7
	3–4 h	9	6.5
	4–5 h	10	7.2
	5–6 h	18	13.0
	6 h or more	84	60.9

The duration of utilization of special education center services.

Min: 2 months, Max: 228 months, Mean: 50.78 months.

There was no statistically significant correlation between the duration of special education service use and the scale scores (*p* > 0.05, Pearson Correlation).

### 3.2 Internalized stigma, mindfulness, resilience, and caregiver burden scores of parents

The total psychological resilience score of the participants was found to be 14.39 ± 4.91 (skewness = −0.674; kurtosis = 0.265), the total internalized stigma score was 65.97 ± 21.38 (skewness = −1.306; kurtosis = 0.724), the total caregiver burden score was 55.59 ± 17.94 (skewness = −1.368; kurtosis = −0.027), and the total mindful awareness score was 52.93 ± 19.46 (skewness = −1.390; kurtosis = 0.083). Examination of the Cronbach’s alpha values of the scales indicates that all scales exhibit an adequate level of reliability. The Brief Resilience Scale (BRS) demonstrated acceptable reliability with a Cronbach’s alpha of 0.764, while the Parents’ Internalized Stigma of Mental Illness Scale (PISMIS), Zarit Caregiver Burden Scale (ZCBS), and Mindful Attention Awareness Scale (MAAS) exhibited excellent reliability, with Cronbach’s alpha values of 0.957, 0.945, and 0.935, respectively, indicating high internal consistency. For further details, refer to Table 2.

**Table 2:** Descriptive statistics for the main variables (BRS, PISMIS, ZCBS and MAAS Scales).

Scales	*N*	Minimum	Maximum	Mean	Std. deviation	Skewness	Kurtosis	Cronbach alpha
Brief Resilience Scale (BRS)	138	5.00	25.00	14.391	4.914	0.265	−0.674	0.764
Parents’ Internalized Stigma of Mental Illness Scale (PISMIS)	138	29.00	108.00	65.97	21.38	0.274	−1.306	0.957
Zarit Caregiver Burden Scale (ZCBS)	138	21.00	88.00	55.59	17.94	−0.027	−1.368	0.945
Mindful Attention Awareness Scale (MAAS)	138	22.00	89.00	52.93	19.46	0.083	−1.390	0.935

### 3.3 Correlation analysis results of internalized stigma, mindfulness, resilience, and caregiver burden of parents

Psychological resilience was negatively correlated with caregiver burden (*r* = −0.681, *p* < 0.001), while caregiver burden was positively correlated with internalized stigma (*r* = 0.742, *p* < 0.001). Mindfulness was negatively correlated with internalized stigma (*r* = −0.734, *p* < 0.001), and psychological resilience was also negatively correlated with internalized stigma (*r* = −0.660, *p* < 0.001). Additionally, psychological resilience was positively correlated with mindfulness (*r* = 0.634, *p* < 0.001). In summary, there were statistically significant correlations among the scale scores (all *p* < 0.001). The results are presented in Table 3.

**Table 3:** Correlation analysis results of Internalized stigma, mindfulness, resilience, and caregiver burden of parents.

Scales	BRS	ZCBS	MAAS	PISMIS
Brief Resilience Scale (BRS)	1			
Zarit Caregiver Burden Scale (ZCBS)	−0.681 (*p* < 0.001)	1		
Mindful Attention Awareness Scale (MAAS)	0.634 (*p* < 0.001)	−0.708 (*p* < 0.001)	1	
Parents’ Internalized Stigma of Mental Illness Scale (PISMIS)	−0.660 (*p* < 0.001)	0.742 (*p* < 0.001)	−0.734 (*p* < 0.001)	1

All correlation coefficients presented in table are statistically significant at *p* < 0.001 (Pearson correlation analysis). all *p* < 0.001.

### 3.4 The differentiation of internalized stigma, mindfulness, resilience, and caregiver burden scores according to socio-demographic variables

The differences in scale scores based on various socio-demographic variables (e.g., age, gender, income level, etc.) were examined using statistical tests, and the results are presented in Table 4. In terms of age, caregiver burden scores differed significantly across age groups (*F* = 2.983, *p* = 0.021). Specifically, parents in the 20–25 and 41 years and above age groups had higher caregiver burden scores. Regarding income level, caregiver burden scores were also found to significantly differ (*F* = 3.039, *p* = 0.040), with parents in the low-income group reporting higher caregiver burden. When analyzed by gender, female parents had significantly higher caregiver burden scores compared to male parents (*t* = 2.096, *p* = 0.038). Similarly, caregiver burden scores were significantly higher among parents who had another child with a disability (*t* = 3.354, *p* = 0.001). No statistically significant differences in caregiver burden scores were observed for other socio-demographic variables (*p* > 0.05). A significant difference was also found between internalized stigma scores and income level (*F* = 3.956, *p* = 0.021), with parents in the low-income group reporting higher internalized stigma than those in the middle-income group. Internalized stigma scores were also significantly higher among parents with another child with a disability (*t* = 3.021, *p* = 0.003). No significant differences in internalized stigma scores were found for the other variables (*p* > 0.05). Resilience scores showed a statistically significant difference based on the presence of another child with a disability. Parents with another child with a disability had significantly lower resilience scores than those who did not (*t* = −3.885, *p* = 0.000). No significant differences in resilience scores were found for other variables (*p* > 0.05).

**Table 4:** Differences in scale (BRS, PISMIS, ZCBS and MAAS) scores according to demographic variables.

Demographic variables	*n*	BRS	ZCBS	MAAS	PISMIS
		Mean ± SD	Mean ± SD	Mean ± SD	Mean ± SD
Age					
20–25	**13**	2.90 ± 0.84	3.23 ± 0.93	3,57 ± 1.61	2,36 ± 0.84
26–30	**20**	3.08 ± 1.17	2.77 ± 1.02	3,36 ± 1.19	2,25 ± 0.78
31–35	**33**	3.03 ± 0.91	2.54 ± 0.75	4,02 ± 1.29	2,03 ± 0.63
36–40	**34**	2.72 ± 1.09	2.92 ± 0.97	3,39 ± 1.38	2,34 ± 0.76
41 and above	**38**	2.75 ± 0.88	3.22 ± 0.92	3,29 ± 1.09	2,40 ± 0.72
*F*=		0.759	2.983	1.706	1.310
*p*=		0.554	0.021*	0.152	0.270
*Post hoc*=			31–35 < 20–25, 31–35 < 41 and above (*p* < 0.05)		
Perceived income level					
Low	**39**	2.69 ± 1.14	3.23 ± 0.94	3.10 ± 1.32	2.54 ± 0.817
Medium	**87**	2.94 ± 0.89	2.77 ± 0.90	3.71 ± 1.22	2.15 ± 0.65
High	**12**	2.98 ± 1.04	2.99 ± 1.03	3.56 ± 1.49	2.29 ± 0.84
*F*=		0.927	3.039	3.050	3.956
*p*=		0.398	0.040*	0.051	0.021*
*Post hoc*=			Low > Medium (*p* < 0.05)		Low > Medium (*p* < 0.05)
Time spent with child daily					
1–2 h	5	3.52 ± 1.16	2.46 ± 1.09	3.93 ± 1.05	2.07 ± 0.82
2–3 h	12	2.83 ± 0.71	2.85 ± 0.90	3.41 ± 1.26	2.34 ± 0.71
3–4 h	9	2.88 ± 0.41	3.02 ± 0.78	3.22 ± 1.37	2.18 ± 0.66
4–5 h	10	2.68 ± 0.84	2.93 ± 0.94	3.01 ± 1.27	2.40 ± 0.74
5–6 h	18	2.61 ± 0.88	3.26 ± 1.06	3.45 ± 1.35	2.40 ± 0.77
6 h or more	84	2.92 ± 1.07	2.87 ± 0.93	3.63 ± 1.30	2.24 ± 0.74
*F*=		0.813	0.769	0.645	0.319
*p*=		0.542	0.573	0.666	0.901
Gender					
Male	29	2.73 ± 0.89	2.84 ± 0.94	3.28 ± 1.32	2.48 ± 0.72
Female	109	2.91 ± 1,00	3.24 ± 0.89	3.59 ± 1.2	2.21 ± 0.73
*t*=		−0.864	2.096	−1.162	1.729
*p*=		0.389	0.038*	0.247	0.086
Employment					
Yes	42	2.77 ± 1.05	3.08 ± 1.00	3.28 ± 1.40	2.38 ± 0.75
No	96	2.92 ± 0.95	2.85 ± 0.91	3.63 ± 1.23	2.22 ± 0.72
*t*=		−0.844	1.272	−1.472	1.108
*p*=		0.400	0.206	0.143	0.270
Disability in other children					
Yes	31	2.30 ± 0.86	3.40 ± 0.85	2.78 ± 1.15	2.17 ± 0.72
No	85	3.04 ± 0.95	2.78 ± 0.92	3.74 ± 1.26	2.61 ± 0.69
*t*=		−3.885	3.354	−3.811	3.021
*p*=		0.000**	0.001*	0.000**	**0.003**
Child’s gender					
Female	48	2.80 ± 1.05	2.90 ± 0.95	3.48 ± 1.49	2.32 ± 0.78
Male	90	2.91 ± 0.94	2.93 ± 0.94	3.55 ± 1.18	2.25 ± 0.71
*t*=		−0.609	−0.184	−0.310	0.534
*p*=		0.544	0.854	0.757	0.594

*F*: ANOVA test; *t*: Independent Samples *T*-Test; *post hoc*: LSD method. *p < 0.05, **p < 0.001. Bold values indicate statistically significant differences (*p* < 0.05).

Lastly, mindfulness scores also significantly differed based on the presence of another child with a disability. Parents with another child with a disability had lower mindfulness scores compared to those without (*t* = 3.021, *p* = 0.003). No other socio-demographic variables showed significant differences in mindfulness scores (*p* > 0.05).

### 3.5 A test of the mediating influence of mindfulness with resilience on the relationship between internalized stigma and caregiver burden in parents

In this study, the nonparametric percentile bootstrap program with Amos bias correction was used to test the significance of the mediating effect. 200 samples were randomly selected from the original sample (*n* = 138) to reduce class I errors in statistical reasoning caused by data. According to the theoretical assumptions of this study, during the modeling process, the internalized stigma of parents was taken as the predictive variable, and resilience with mindfulness was taken as the intermediary variable. Caregiver burden is used as the effect variable (dependent variable) to establish the intermediary effect structural equation model, and the maximum likelihood ratio method is used to establish the correlation between residuals according to the correction indicators given by the model, so as to appropriately improve the model fitting, as shown in Figure 1. Each fitting index of the structural equation model meets the ideal standard, and the model is well adapted, as shown in Table 5.

**Table 5:** Structural equation model (SEM) fit indices.

Project	*χ* ^2^	CMIN/df	GFI	AGFI	RFI	NFI	CFI	IFI	TLI	RMSEA
Modified fitting index	1878	1.682	0.588	0.558	0.608	0.823	0.801	0.803	0.793	0.071
Acceptable standards		≤3	≥0.90 (Good), 0.80–0.89 (Moderate)	≥0.90 (Good), 0.80–0.89 (Moderate)	≥0.90 (Good), 0.80–0.89 (Moderate)	≥0.90 (Good), 0.80–0.89 (Moderate)	≥0.90 (Good), 0.80–0.89 (Moderate)	≥0.90 (İyi uyum), 0.80–0.89 (Moderate)	≥0.90 (İyi uyum), 0.80–0.89 (Moderate)	≤0.08 (Good)

CMIN/df, maximum likelihood ratio *χ*^2^ values/degrees of freedom; GFI, goodness of fit index; AGFI, adjusted goodness of fit index; RFI, relative fitness index; NFI, standard fit index; CFI, comparative fit index; IFI, value added index; TLI, nonstandard fit index; RMSEA, Mean square sum square root of progressive residuals.

Given the exploratory nature of the study and the modest sample size, model fit indices should be interpreted with caution.

As shown in Table 5, the structural equation model demonstrated acceptable to moderate fit to the data. While some fit indices (e.g., RMSEA) met commonly accepted thresholds, others fell slightly below optimal cut-off values. Given the complexity of the proposed mediation model and the modest sample size, the overall model fit was considered adequate for exploratory mediation analysis (Byrne, 2001).

### 3.6 Estimated parameters and 90% confidence intervals for internalized stigma, psychological resilience, caregiver burden, and mindfulness of parents

**Figure 2:**
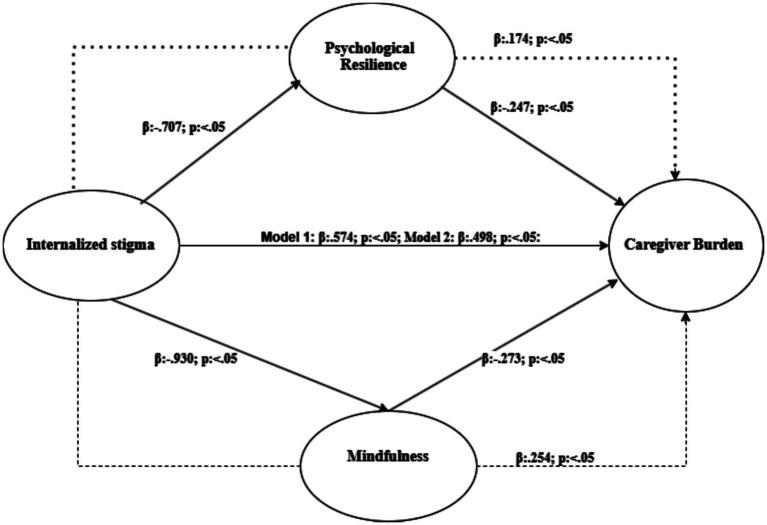
Mediating model of mindfulness and psychological resilience in the relationship between internalized stigma and caregiver burden among caregivers.

This study examined the mediating effects of psychological resilience and mindfulness on the relationship between internalized stigma and caregiver burden among parents of children with ASD. The results of the mediation analysis revealed significant associations among internalized stigma, psychological resilience, mindfulness, and caregiver burden (Figure 2, Table 6).

**Table 6:** Total, direct, and indirect effects of each path in this model.

Variables	Estimate (*ß*)	SE	C.R.	*p*	Upper bounds	Lower bounds
Model 1						
Direct (internalized → caregiver burden stigma)	0.574	0.112	6.675	***	0.835	0.294
Indirect (internalized → caregiver burden via stigma psychological resilience)	0.174	0.106	–	***	0.376	0.069
Total (internalized → caregiver burden “without stigma mediatör”)	0.748	0.128	–	**0.010**	1.005	0.551
Internalized → psychological resilience stigma	−0.707	0.113	−6.257	***	−0.499	−0.934
Psychological resilience → caregiver burden	−0.247	0.079	3.121	**0.002**	−0.120	−0.478
Model 2						
Direct (internalized → caregiver burden stigma)	0.498	0.112	6.675	***	0.835	0.294
Indirect (internalized → caregiver burden via stigma mindfulness)	0.254	0.095	–	**0.010**	0.424	0.106
Total (internalized → caregiver burden “without stigma mediatör”)	0.752	0.132	–	**0.010**	1.000	0.555
Internalized → mindfulness stigma	−0.930	0.141	−6.604	***	−0.698	−1.185
Mindfulness → caregiver burden	−0.273	0.086	−3.178	**0.001**	−0.107	−0.449

***p < 0.001. Bold values indicate statistically significant mediation effects.

In the first phase of the analysis (Model 1), the mediating role of psychological resilience in the relationship between internalized stigma and caregiver burden was examined. The direct effect of internalized stigma on caregiver burden was positive and statistically significant [*β* = 0.574, SE = 0.112, C.R. = 6.675, *p* < 0.05, 90% CI (0.294, 0.835)], supporting Hypothesis 1.

Psychological resilience was found to negatively and significantly predict caregiver burden [*β* = −0.247, SE = 0.079, C.R. = −3.121, *p* = 0.002, 90% CI (−0.478, −0.120)], which supports Hypothesis 2. Additionally, internalized stigma negatively predicted psychological resilience [*β* = −0.707, SE = 0.113, C.R. = −6.257, *p* < 0.001, 90% CI (−0.934, −0.499)], confirming Hypothesis 3.

In the final step of this phase, internalized stigma and the proposed mediator (psychological resilience) were included in the model simultaneously to examine their effects on caregiver burden. The effect of internalized stigma on caregiver burden remained significant but decreased (*β* = 0.174, *p* ≤ 0.05), while the effect of psychological resilience on caregiver burden also remained significant (*β* = −0.247, *p* < 0.05). This finding indicates a partial mediating effect of psychological resilience in the relationship between internalized stigma and caregiver burden, supporting Hypothesis 4. In other words, the inclusion of psychological resilience in the model reduced the strength of the direct effect of internalized stigma on caregiver burden.

In the second phase of the analysis (Model 2), the mediating role of mindfulness was tested in the relationship between internalized stigma and caregiver burden. Internalized stigma negatively and significantly predicted mindfulness [*β* = −0.930, SE = 0.141, C.R. = −6.604, *p* < 0.001, 90% CI (−1.185, −0.698)], supporting Hypothesis 5.

Mindfulness, in turn, negatively and significantly predicted caregiver burden [*β* = −0.273, SE = 0.086, C.R. = −3.178, *p* = 0.001, 90% CI (−0.449, −0.107)], supporting Hypothesis 6. In the final step of this model, both internalized stigma and mindfulness were included simultaneously to assess their impact on caregiver burden. Internalized stigma maintained a significant but reduced effect on caregiver burden (*β* = 0.254, *p* ≤ 0.05), while the effect of mindfulness also remained significant (*β* = −0.273, *p* < 0.005).

Additionally, internalized stigma had an indirect effect on caregiver burden through mindfulness [*β* = 0.254, *p* < 0.05, 90% CI (0.106, 0.424)], confirming a partial mediating role of mindfulness in the relationship between internalized stigma and caregiver burden. These results provide empirical support for Hypothesis 7.

## 4 Discussion

The study focused on the primary caregivers of children with ASD attending special education and rehabilitation centers, and revealed that caregiver burden, internalized stigma, psychological resilience, and mindfulness levels significantly differed based on certain demographic characteristics.

In the literature, several studies have indicated that parental age has an impact on caregiver burden. In a study conducted by Emir et al. (2020) with parents of children with special needs, it was reported that caregiver burden scores increased with age. Özok Bulut et al. (2024) found that younger parents in the 20–30 age group had higher caregiver burden scores. In the present study, caregiver burden scores were found to be higher among both younger parents aged 20–25 and older parents aged 41 and above. This finding may be associated with the lack of experience and increased stress levels among younger parents, as well as the greater likelihood of physical and psychological fatigue experienced by older parents.

In the current study, caregiver burden scores of female parents were found to be higher than those of male parents. There are other studies in the literature that support this finding (Gümüş and Kaçan, 2024). In the study by Patel et al. (2022), mothers as caregivers were found to experience a significantly higher level of caregiver burden. This was attributed to the fact that mothers are typically the primary caregivers who assume full responsibility for caregiving and spend the most time with the child. Other studies have also demonstrated that the caregiver burden predominantly falls on mothers, who are solely responsible for meeting the child’s daily needs, which in turn leads to increased stress (Abdulla and Al Kurwi, 2020; Minhas et al., 2015). The findings of the present study are consistent with these results. The higher caregiver burden experienced by women compared to men may reflect traditional gender roles, wherein caregiving responsibilities are predominantly assumed by women. This situation may also be explained by the fact that women often serve as the primary caregivers and therefore bear a greater share of the physical and emotional burden.

Another key finding of our study is that parents with lower income levels reported higher caregiver burden and internalized stigma, suggesting that economic hardship exacerbates the challenges of the caregiving process. Socioeconomic status plays a crucial role in helping parents of children with ASD cope with the difficulties of daily life (Alwhaibi et al., 2020). Parents with higher household incomes are more likely to access psychologists, therapists, and educators for both themselves and their children’s special needs, which in turn enhances their perceived social support (Al-Farsi et al., 2013). However, low income may prevent parents from benefiting sufficiently from such support systems. This suggests that economic disadvantages may reinforce feelings of stigma by increasing social isolation and stress levels.

Additionally, parents who have an additional child with a disability reported higher levels of caregiver burden and internalized stigma, along with lower levels of psychological resilience and mindfulness. This suggests that having more than one child with special needs may impose additional stress and burnout on parents, and the increased responsibilities may negatively impact their psychological resilience and level of mindfulness.

This study further examined the mediating roles of mindfulness and psychological resilience in the relationship between internalized stigma and caregiver burden among parents of children with autism spectrum disorder using structural equation modeling. The findings provided preliminary support for the proposed mediation pathways; however, the overall model demonstrated moderate rather than optimal fit to the data. Therefore, the results should be interpreted with caution and considered exploratory in nature, particularly given the cross-sectional design and the complexity of the psychosocial constructs examined.

Overall, the findings revealed significant relationships between internalized stigma, psychological resilience, mindfulness, and caregiver burden, highlighting important psychological mechanisms underlying the caregiving process. The high levels of statistically significant correlations among the study variables (all *p* < 0.001) are consistent with previous studies (Gümüş and Kaçan, 2024; Wang et al., 2024; Yip and Chan, 2022; Salleh et al., 2024; Xing et al., 2025). However, the magnitude of these associations warrants careful interpretation. The high correlation coefficients observed in this study may be explained by the theoretical proximity between internalized stigma, mindfulness, and psychological resilience. However, these relationships do not imply that the constructs are identical or interchangeable. Due to the cross-sectional design of the study, the findings should be interpreted at a relational rather than a causal level. In particular, the intense intertwinement of psychosocial processes among caregivers of children with autism spectrum disorder may have contributed to the strong associations observed among these constructs.

The negative relationship between psychological resilience and caregiver burden suggests that parents with higher levels of resilience are better able to cope with stress and therefore perceive a lower caregiving burden. This implies that psychologically resilient individuals may possess more effective coping skills in managing stressful caregiving situations. This finding aligns with the protective role of psychological resilience emphasized in studies such as those by Doğan (2015) and Rasoulpoor et al. (2023). Other findings in the literature similarly underscore the protective function of resilience in highly stressful contexts such as chronic caregiving (Bekhet et al., 2012).

The positive correlation between caregiver burden and internalized stigma suggests that as caregiving responsibilities increase, parents may be more prone to internalizing societal stigma. This may indicate that the psychological pressure associated with caregiving can trigger feelings such as inadequacy, guilt, or shame, thereby intensifying the sense of stigma (Mak and Kwok, 2010). Another variable found to be negatively correlated with internalized stigma is mindfulness, which may serve as a protective psychological buffer in this process. Parents with higher levels of mindfulness tend to adopt a more present-focused and non-judgmental attitude, making them less susceptible to negative self-perceptions. Mindfulness practices may play this protective role by enhancing self-acceptance and emotional regulation skills. Similarly, psychological resilience was also found to be negatively associated with internalized stigma. This finding suggests that psychologically resilient parents are less affected by external negative judgments, potentially due to their capacity to maintain a sense of meaning and purpose while coping with adversity (Connor and Davidson, 2003). The negative impact of internalized stigma on both psychological resilience and mindfulness indicates that stigma extends beyond social perceptions and also undermines individuals’ internal worlds and mental flexibility. The mediating role of mindfulness is consistent with stigma coping models proposed by Chan et al. (2018) and Wang et al. (2024).

Another key finding of our study is that, based on the results of two mediation models tested using structural equation modeling, both psychological resilience and mindfulness partially mediate the relationship between internalized stigma and caregiver burden. As emphasized in previous research, resilience and mindfulness are often regarded as important compensatory mechanisms that buffer individual psychological responses when faced with challenging circumstances, and they are considered moderators in the relationship between stressors and psychological outcomes (Brown-Iannuzzi et al., 2014; Tang et al., 2021; Yip and Chan, 2022; Salleh et al., 2024; Xing et al., 2025; Rasoulpoor et al., 2023). These findings underscore the importance of enhancing psychological resilience and mindfulness, particularly for parents experiencing high levels of caregiver burden and internalized stigma. Intervention programs aimed at increasing resilience and developing mindfulness skills may positively impact parents’ well-being and caregiving experiences. Furthermore, the results suggest that such interventions should not only target external stressors but also focus on strengthening parents’ internal resources. Future research should employ longitudinal designs to determine the directionality of these relationships and assess the effectiveness of specific psychosocial interventions in reducing stigma and caregiver burden.

## 5 Limitations

This study has several limitations. It was conducted with a sample of 138 parents receiving services from five special education and rehabilitation centers in a single province in Turkey. The small and non-representative nature of the sample limits the generalizability of the findings to other geographical regions. To improve generalizability, future research should involve larger sample sizes and include parents from centers located in other provinces. Additionally, the use of self-reported measures may introduce subjectivity and potential bias, which could further limit the validity and generalizability of the results.

The findings provided preliminary support for the proposed mediation pathways; however, the overall model demonstrated moderate rather than optimal fit to the data. Therefore, the results should be interpreted with caution and considered exploratory in nature, particularly given the cross-sectional design and the modest sample size, as well as the complexity of the psychosocial constructs examined. Future studies employing longitudinal designs and alternative modeling approaches are recommended to further validate the proposed mediation pathways.

Another limitation of this study is that the sample included only parents of children attending Special Education and Rehabilitation Centers. Parents of children with ASD who attend mainstream schools with inclusive education support may experience stigma, caregiver burden, and psychosocial processes differently. Therefore, the generalizability of the findings to all families of children with ASD may be limited.

Another limitation of this study is that the severity of autism spectrum disorder and the presence of comorbid conditions (e.g., intellectual disability, attention-deficit/hyperactivity disorder, or anxiety disorders) were not assessed. These factors may influence caregiver burden, internalized stigma, psychological resilience, and mindfulness. Therefore, future studies should incorporate standardized assessments of ASD severity and comorbidities to better elucidate their potential impact on caregiving-related psychosocial outcomes.

## 6 Conclusion

These research findings indicate that the levels of caregiver burden and internalized stigma among parents of children with Autism Spectrum Disorder are influenced by demographic factors such as age, gender, income level, and the presence of other children with disabilities. In particular, younger parents, women, those with low income, and those with multiple children with disabilities appear to be at higher risk. Psychosocial support programs can be developed specifically for parents in these high-risk groups, while financial assistance and counseling services may be strengthened for low-income families. Awareness-raising initiatives that promote the sharing of caregiving responsibilities within the family could help reduce the burden on women. Additionally, tailored counseling and therapy services for parents of multiple children with disabilities, as well as guidance programs for young parents, could teach effective strategies for alleviating caregiver burden.

Psychological resilience is often regarded as an important compensatory factor that buffers individual psychological responses when faced with adversity and facilitates coping with challenging circumstances. It is also considered a moderator in the relationship between stigma and psychological outcomes, such as increased caregiver burden.

Our findings highlight the link between internalized stigma and negative outcomes—such as elevated caregiver burden—among parents of children with ASD, and they demonstrate the potential protective roles of mindfulness and psychological resilience against such outcomes. Future interventions aimed at reducing caregiver burden in parents of children with ASD may be improved by incorporating resilience-building and mindfulness training programs.

## Data Availability

The raw data supporting the conclusions of this article will be made available by the authors, without undue reservation.
